# Transcriptome Revealed the Macrophages Inflammatory Response Mechanism and NOD-like Receptor Characterization in Siberian Sturgeon (*Acipenser baerii*)

**DOI:** 10.3390/ijms24119518

**Published:** 2023-05-30

**Authors:** Defang Chen, Yinqiu Chen, Lu Lu, Hao Zhu, Xin Zhang, Xiaoli Huang, Zhiqiong Li, Ping Ouyang, Xiaoli Zhang, Liangyu Li, Yi Geng

**Affiliations:** 1 Aquaculture Department, College of Animal Science and Technology, Sichuan Agricultural University, Chengdu 611130, China; 2 Research Center of Aquatic Animal Diseases, College of Veterinary Medicine, Sichuan Agricultural University, Chengdu 611130, China; 3 Institute of Fisheries Research, Chengdu Academy of Agricultural and Forestry Sciences, Chengdu 611130, China

**Keywords:** *Acipenser baerii*, LPS, transcriptome, inflammatory response, cytokines, NOD-like receptor

## Abstract

Nucleotide-binding and oligomerization domain-like receptors (NOD-like receptors, NLRs) can regulate the inflammatory response to eliminate pathogens and maintain the host’s homeostasis. In this study, the head kidney macrophages of Siberian sturgeon were treated with lipopolysaccharide (LPS) to induce inflammation by evaluating the expression of cytokines. The high-throughput sequencing for macrophages after 12 h treatment showed that 1224 differentially expressed genes (DEGs), including 779 upregulated and 445 downregulated, were identified. DEGs mainly focus on pattern recognition receptors (PRRs) and the adaptor proteins, cytokines, and cell adhesion molecules. In the NOD-like receptor signaling pathway, multiple NOD-like receptor family CARD domains containing 3-like (NLRC3-like) were significantly downregulated, and pro-inflammatory cytokines were upregulated. Based on the transcriptome database, 19 NLRs with NACHT structural domains were mined and named in Siberian sturgeon, including 5 NLR-A, 12 NLR-C, and 2 other NLRs. The NLR-C subfamily had the characteristics of expansion of the teleost NLRC3 family and lacked the B30.2 domain compared with other fish. This study revealed the inflammatory response mechanism and NLRs family characterization in Siberian sturgeon by transcriptome and provided basic data for further research on inflammation in teleost.

## 1. Introduction

Inflammation plays an essential role in disease response, courses of disease development, and excessive inflammation detrimental to the host homeostasis of vertebrates [1]. After microorganisms infection, macrophages and granulocytes can enhance respiratory bursts and promote the release of active oxygen and nitrogen ions; they have been considered the core cells of the inflammatory response [2]. The macrophages of the host can activate nuclear factor kappa-B (NF-κB) to promote the release of pro-inflammatory cytokines interleukin-1β (IL-1β), tumor necrosis factor-α (TNF-α), interleukin-6 (IL-6), and interleukin-8 (IL-8) after pattern recognition receptors (PRRs) recognize pathogens or pathogen-associated molecular patterns (PAMPs) [3]. The cytokines improve the host’s immune ability, eliminate pathogens, and up-regulate the anti-infection function by activating the different lymphocytes [4]. Therefore, inflammatory response mediated by PRRs plays an essential role in maintaining homeostasis.

Nucleotide-binding and oligomerization domain-like receptors (NOD-like receptors, NLRs) are one of the most essential PRRs located in the cytoplasm [5]. NLRs have the conserved nucleotide-binding and oligomerization domain (NACHT) structures from insects to mammals, and the R proteins with similar domains are also found in plants [6,7]. A previous study showed that NLRs play a critical role in innate immunity by recognizing ligands and mediating inflammatory response [8]. So far, a total of 22 NLRs have been identified in humans (*Homo sapiens*), including CIITA of the NLR-A subfamily, NAIP of the NLR-B subfamily, NOD1 (NLRC1), NOD2 (NLRC2), NLRC3, NLRC4, NLRC5, and NLRX of the NLR-C subfamily, and NLRP1-NLRP14 of the NLRP subfamily [9]. Compared with mammals, the NLRs of teleost are polymorphism due to chromosome replication events. The NLRC3 has many expansions whose structure possessed PYD, CARD, FISNA, and B30.2 domains or missing the LRRs domain [10]. Therefore, the naming method for NLRs of mammals is not applicable in lower vertebrates.

In recent years, based on the genome and transcriptome database, NLRs of teleost have been reported in zebrafish (*Danio rerio*) [11], channel catfish (*Ictalurus punctatus*) [12], miiuy croaker (*Miichthys miiuy*) [13], grass carp (*Ctenopharyngodon Idella*) [14], black rockfish (*Sebastes schlegeli*) [15], Chinese tongue sole (*Cynoglossus semilaevis*) [16], and turbot (*Scophthalmus maximus* L.) [17]. Piscine NLRs were classified into four subfamilies, NLR-A, NLR-B, NLR-C, and other NLRs. The NLR-A includes the mammalian NLR-A subfamily (CIITA) and NLR-C subfamily (NOD1, NOD2, and NLRC5). NLR-B subfamily include NLR-B1, NLR-B2, NLR-B3, NLR-B4, and NLR-B5. NLR-C subfamily has the largest number of genes, including NLRC3 and NLRC3-like with gene expansion. Other NLRs include the genes such as NLRX, APAF, NWD1, and NWD2 [17]. However, there are some differences in the naming of different species. The NLRs with the B30.2 domain in grass carp were named NLR-B30.2, and NLRX was classified as an NLR-A subfamily [14]. All NLRs of the Chinese tongue sole containing the FISNA domain were named NLR-C, and NOD3/NLRC3 and NOD5/NLRX1 belong to the NLR-A subfamily [16]. Unlike the Teleostei, the Acipenseriformes are independent at the early stage of differentiation of the Scleroichthys. The NLRs genes of sturgeon have only been slightly annotated in the database, while the studies on identification, function, and mechanism are scarce [18]. Therefore, exploring the structure of NLRs in Acipenseriformes provide an important reference for understanding the origin and evolution of innate immunity.

This study used the macrophages of Siberian sturgeon to explore the inflammatory response mechanism by measuring cytokines and transcriptome sequencing after LPS treatment. The NLRs of the sturgeon were analyzed with phylogenetic trees constructed and the structure domain predicted. This study enriches the insight into the function of innate immunity in vertebrates and provides the regulator target in the inflammatory response in sturgeon.

## 2. Results

### 2.1. The Expression of Cytokines after LPS Treatment

**Figure 1:**
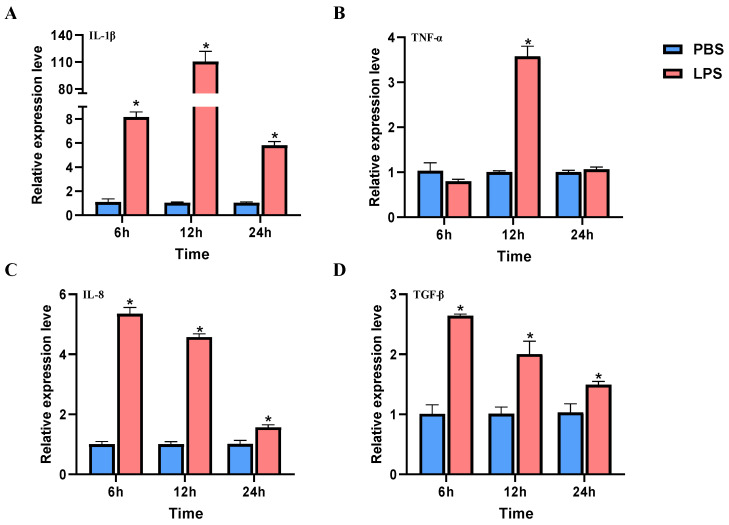
Inflammatory cytokines expression after LPS treatment in head kidney macrophages. (**A**–**D**) Relative expression level of *IL-1β* (**A**), *TNF-α* (**B**), *IL-8* (**C**) and *TGF-β* (**D**) at 6 h, 12 h and 24 h. * indicates significant difference.

After LPS treatment of macrophages, the results showed that the mRNA expression of *IL-1β* was significantly upregulated at 6 h, 12 h, and 24 h, and the highest expression level at 12 h was 110.40 times that of the control. The relative expression level of *TNF-α* was significantly upregulated at 12 h, which was 3.57 times higher than the control group. Compared with PBS treatment, the relative expression levels of *IL-8* and *TGF-β* were significantly upregulated at 6 h, 12 h, and 24 h with a gradually decreasing trend (Figure 1).

### 2.2. Raw Sequence and De Novo Assembly

After de novo assembly, the clean reads were 45,822,444, 43,771,700, 41,679,648, and 42,727,782 in the PBS group and 40,638,064, 42,547,796, 41,011,368, and 52,064,962 in the LPS group, respectively. The amounts of clean reads in different groups are shown in Table 1. The clean reads were retained with Q30 > 93.8%, and the error rate was <0.0255%. The data were of high quality and could be used for the subsequent annotation analysis.

### 2.3. Functional Annotation

**Figure 2:**
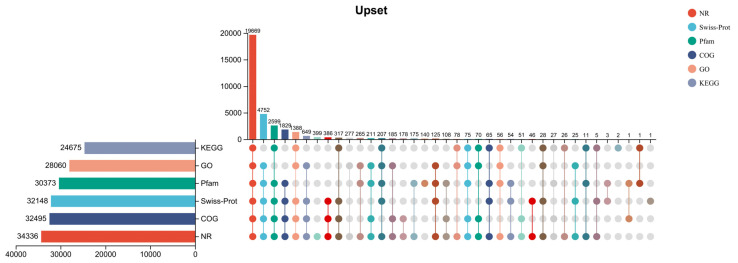
Upset of unigenes annotations. The horizontal bar chart on the left represents the statistics of gene expression quantities annotated in six different databases. In the right chart, a single point represents the annotated genes in the database, and the lines between points represent the intersection of different databases. The vertical bar chart represents the number of genes in the corresponding database, respectively.

Compared with different databases, the results showed that 34,484 (96.68%) unigenes have annotated in at least one database (Figure 2). Moreover, 34,336 (96.27%), 32,495 (91.10%), 32,148 (90.13%), 30,373 (85.15%), 28,060 (78.67%), and 24,675 (69.18%) were annotated in NR, COG, Swiss-prot, Pfam, GO, and KEGG, respectively (Table 2).

### 2.4. Principal Component Analysis (PCA) and Identification of DEGs

**Figure 3:**
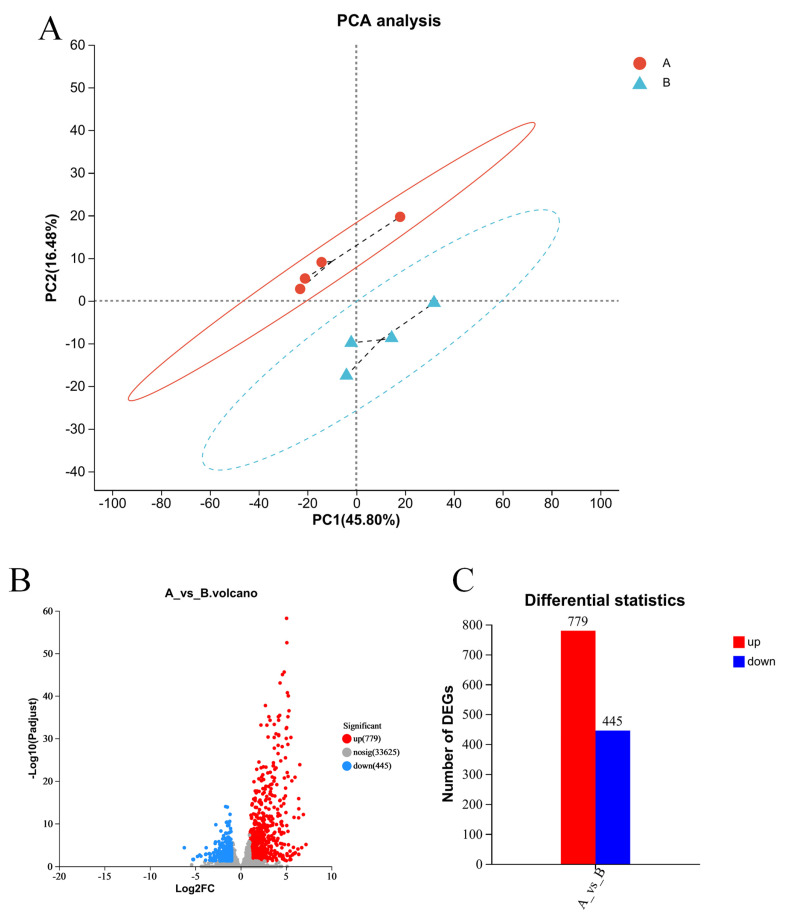
Analysis of principal component analysis and differentially expressed genes. (**A**) Analysis of principal component analysis. The horizontal axis represents the contribution of PC1 to distinguishing samples, while the vertical axis represents the contribution of PC2 to distinguishing samples. (**B**) Analysis of differentially expressed genes. The red dot represents significantly upregulated genes, the blue dot represents significantly downregulated genes, and the gray dot represents non-significantly differentially expressed genes. (**C**) Quantification of B-graph results.

The PCA showed that the PBS and LPS groups were divided into two clusters. Between the PBS and LPS treatment, 1224 unigenes were differentially expressed after LPS treatment macrophages of Siberian sturgeon, including 779 upregulated unigenes and 445 downregulated unigenes (Figure 3).

### 2.5. GO and KEGG Enrichment Analysis

**Figure 4:**
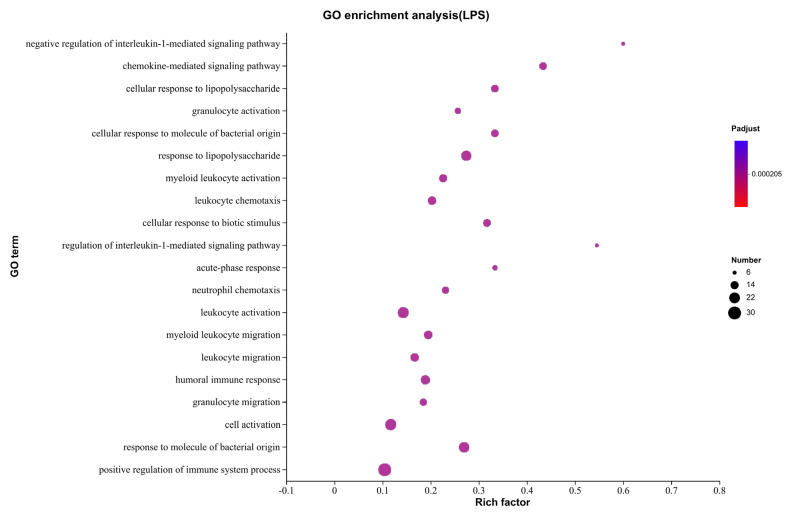
Gene function enrichment analysis in GO database. The *y* axis is the GO term, and the *x* axis represents the significance level of enrichment, which corresponds to the height of the column.

GO database enrichment analysis showed that DEGs were mainly enriched in biological processes, including the negative regulation of the interleukin-1-mediated signaling pathway, chemokine-mediated signaling pathway, cellular response to lipopolysaccharide, granulocyte activation, cellular response to molecule of bacterial origin, and response to lipopolysaccharide. The rich factor of negative regulation of the interleukin-1-mediated signaling pathway was 0.6, followed by the regulation of the interleukin-1-mediated signaling pathway and the chemokine-mediated signaling pathway, which were 0.55 and 0.43, respectively (Figure 4).

**Figure 5:**
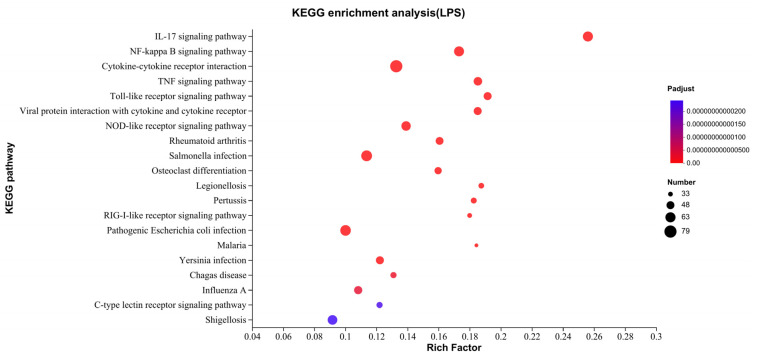
Gene function enrichment analysis in KEGG signaling pathway. KEGG pathway enrichment classification, the *x* axis is the rich factor, which means the ratio of the sample number to the background number, and the *y* axis is the name of the pathway.

After LPS treatment, the results of KEGG enrichment showed that the signaling pathway related to cytokines production was significantly enriched, including the IL-17 signal pathway, cytokine receptor interaction, NF-κB signal pathway, and TNF signal pathway. In addition, PRRs-related pathways were also enriched, such as the Toll-like receptor signal pathway, NOD-like receptor signal pathway, and RIG-I-like receptor signal pathway (Figure 5).

### 2.6. The Analysis of the NOD-like Receptor Signaling Pathway

**Figure 6:**
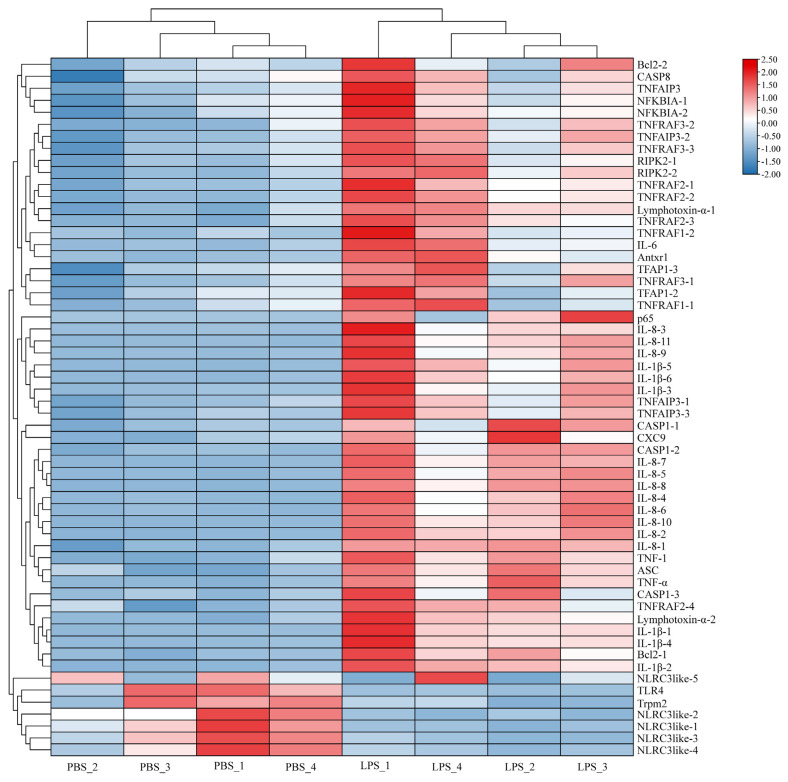
The heatmap of DEGs related to NOD-like receptor signaling pathway in Siberian sturgeon macrophages. The red and blue colors represent up- and downregulated in the PBS and LPS groups, respectively.

Based on the NR annotation and KEGG enrichment analysis, a total of 58 genes of NOD-like receptor signaling pathway, including tumor necrosis factor family, interleukin, chemokine, and chemokine receptor, PRRs, and adaptor protein, nuclear transcription factor, and caspases (Figure 6).

### 2.7. The Analysis of Sturgeon NLR

**Figure 7:**
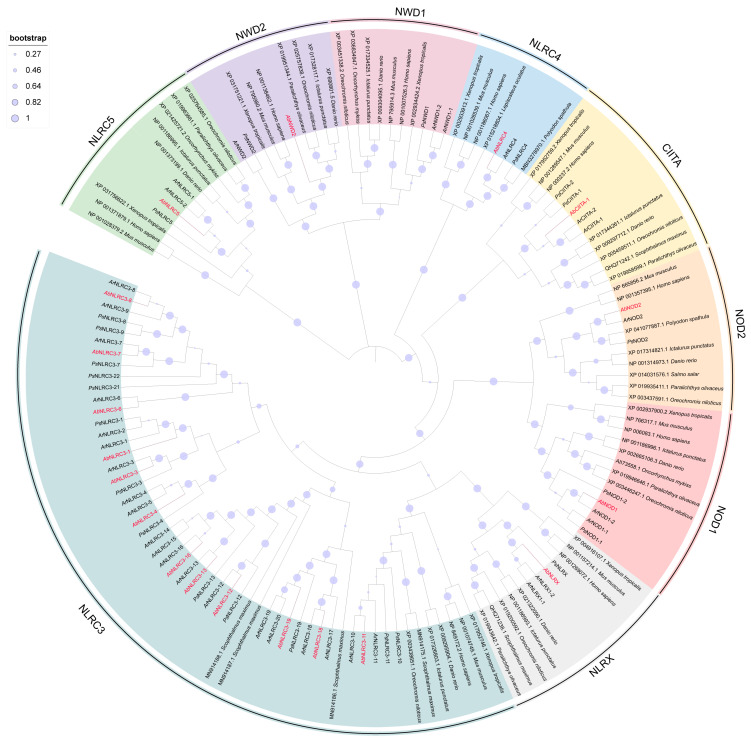
Phylogenetic tree of NLRs genes from sterlet, paddlefish, and Siberian sturgeon.

According to the analysis of the genome database of sterlet and paddlefish, a total of 35 NLRs could encode a conservative NACHT domain. Based on the classification of fish NLRs, they were divided into three subfamilies, namely NLR-A, NLR-C, and other NLRs. NLR-A includes 8 genes including NOD1-1, NOD1-2, NOD2, CIITA-1, CIITA-2, NLRC4, NLRC5-1, and NLRC5-2, NLR-C includes 22 genes, including NLRC3-1, NLRC3-2, NLRC3-3 to NLRC3-22, and other NLRs include 5 genes, including NLRX1-1, NLRX-2, NWD1-1, NWD1-2, and NWD2. The results of the phylogenetic tree showed that the relationship between NOD1, NOD2, CIITA, and NLRC5 of different species was relatively close (Figure 7).

To obtain the NLRs sequence of Siberian sturgeon, the transcriptome database of Siberian sturgeon was constructed based on TBtools. The NLRs sequences of sterlet and paddlefish were compared one by one, and the sequences with the highest homology were obtained for phylogenetic tree analysis. The results showed that there were 19 NLRs in Siberian sturgeon, including 5 NLR-A family members, namely *Ab*NOD1, *Ab*NOD2, *Ab*CIITA, *Ab*NLRC4, and *Ab*NLRC5. A total of 12 NLR-C family members, including *Ab*NLRC3-1, *Ab*NLRC3-3, *Ab*NLRC3-4, *Ab*NLRC3-6, *Ab*NLRC3-7, *Ab*NLRC3-8, *Ab*NLRC3-11, *Ab*NLRC3-12, *Ab*NLRC3-13, *Ab*NLRC3-16, *Ab*NLRC3-18, and *Ab*NLRC3-19, as well as 2 other NLR family members, *Ab*NLRX and *Ab*NWD2 (Figure 7).

**Figure 8:**
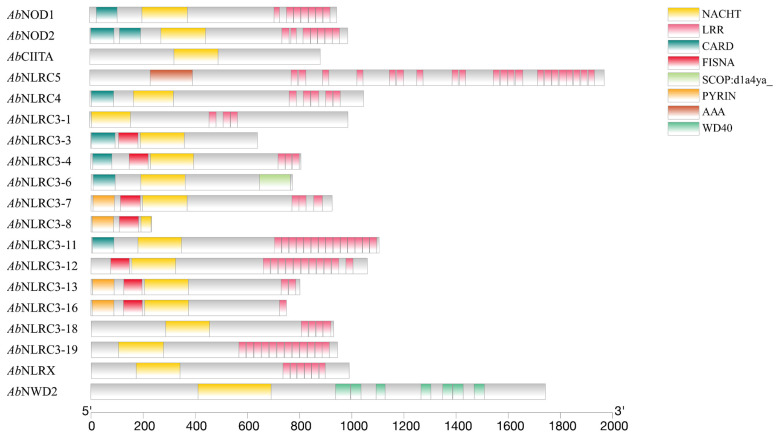
NLRs domain prediction of Siberian sturgeon.

SMART prediction showed that *Ab*NOD1, *Ab*NOD2, and *Ab*NLRC4 of the NLR-A family had the conservative CARD-NACHT-LRRs domain type. *Ab*CIITA only had the NACHT and no LRRs. *Ab*NLRC5 was AAA-LRRs, similar to the sterlet and paddlefish. There were 12 NLR-C family genes in Siberian sturgeon, which have the characteristics of expansion of NLRC3 in teleost. *Ab*NLRC3-1, *Ab*NLRC3-18, and *Ab*NLRC3-19 were NACHT-LRRs types. The effect domains of *Ab*NLRC3-3, *Ab*NLRC3-4, *Ab*NLRC3-6, and *Ab*NLRC3-11 were CARD, and the effect domains of *Ab*NLRC3-7, *Ab*NLRC3-8, *Ab*NLRC3-13, and *Ab*NLRC3-16 were PYRIN (Figure 8).

## 3. Discussion

LPS is the PAMPs of Gram-negative bacteria and causes host inflammatory response, which has been widely used in the construction of inflammatory models and related immunology research. In this study, *IL-1β*, *TNF-α*, *IL-8,* and *TGF-β* were significantly induced at 12 h after LPS treatment, indicating that LPS stimulated the inflammatory response of macrophages of Siberian sturgeon. In bony fish, *IL-1β*, *IL-6*, *CCL2*, and *TNF-α* were significantly upregulated in the head kidney macrophages of large yellow coraker (*Pseudosciaena crocea*) [19] and grass carp [20,21] after LPS treatment in vitro, respectively. Transcriptome can reveal the transcription of mRNA expression level and has been widely used to research the mechanism of cancer [22], genetic disease [23], pathogen infection [24], and stress [25] of the host. To further explore the mechanism of the LPS-induced inflammatory response of Siberian sturgeon, the macrophages was analyzed at 12 h by transcriptome sequencing. Through the identification of DEGs and functional enrichment analysis, the results showed that inflammation-related pathways were significantly enriched, such as the Toll-like receptor signal pathway, NOD-like receptor signal pathway, chemokine–chemokine receptor interaction, and NF-κB signal pathway. Consistent with this study, after intraperitoneal injection of LPS from *Aeromonas hydrophila* or *Escherichia coli*, the transcriptome of the spleen showed that the KEGG signal pathway was enriched in Toll-like receptor signal pathway, NOD-like receptor signal pathway, and chemokine–chemokine receptor interaction [26]. Therefore, LPS can activate the host inflammatory response by inducing the expression of cytokines in the macrophages of Siberian sturgeon.

PRRs are an essential part of the innate immune system to resist the infection of pathogens by recognizing the PAMPs and mediating the immune response to eliminate the pathogen [27]. The PRRs of TLRs [28], NLRs [29], RLRs [30], DNA sensors [31], and CLRs [32] of teleost have been widely studied in function and mechanism in response to pathogens. Different from mammals, NLRs of low vertebrates show a greatly amplified by molecular biology and phylogenetic analysis with the domains of PYRIN, CARD, FISNA, LRRs, or B30.2 [10]. In the Cyclostomata, lamprey (*Lampetra japonicum*), as the representative species of jawless vertebrates only had 2 NLR-A (NODa and NODb) and 7 NLR-C (NLRC3a-NLRC3g) identified in the genome without PYD, and further analysis speculated that the non-CARD NODa and NODb were the common ancestors of the jawless vertebrates NOD1 and NOD2 [33]. NLRs in the purple sea urchin (*Heliocidaris erythogama*) had expanded to an extensive family with 203 NLRs identified. The structure of most NLR proteins was composed of the N-terminal CARD, central NACHT domain, and C-terminal LRRs [34]. In the bony fish, NLRs were also expanded with species-specific. A total of 65 and 29 NLRs with highly conserved NACHT were identified in grass carp and turbot, respectively [14,17]. A total of 23 NLRs were identified in black rockfish, including conserved NOD1, NOD2, NLRC5, and NLRX1, and 15 NLRC3 with gene expansion without PYD or B30.2 [15]. In the present study, the NLRs analysis of Siberian sturgeon shows the specific NLRC4 gene, two isoforms of NOD1 and without containing the B30.2 domain compared with other fish, which also suggests that the sturgeon is different from the Teleostei. The previous study showed that the sturgeon is one of the oldest and earliest vertebrates based on molecular analysis, and the sturgeon had a strong disease resistance after bacterial infection [18]. This study indicated that the unique NLRs may participate in the innate immune defense.

In this study, the multiple NLRC3-like were significantly downregulated after LPS treatment. A previous study confirmed that NLRC3 of mammals belonged to the negative regulatory NLR on inflammation. The protein structure consists of the typical domain of the NACHT and LRRs, and without the effector domain in N-terminal [35]. In mammals, NLRC3 attenuated Lys63 (K63)-linked ubiquitination of TNF receptor-associated factor 6 (TRAF6) and inhibited the activation of the NF-κB [35,36]. However, NLRC3 of teleost has been proven to have a different function in regulating the inflammatory response because of the NLRC3 gene expansion [10]. In flounder, RNA interference with NLRC3 (FISNA-NACHT-LRR-B30.2) downregulated the expression of LPS-induced *IL-1β*, *IL-8*, and *TNF-α*. A study on Nile tilapia showed that the NF-κB signaling pathway was activated after overexpression of NLRC3 (CARD-NACHT-LRR). On the contrary, zebrafish NLRC3-like 1 (FISNA-NACHT-LRR) played a negative role in inflammatory regulation by targeting the RIPK2 and inhibiting the recruitment of NOD1-RIPK2 [10]. NLRC3-like (PYD-NACHT)-deficient zebrafish induced the expression of pro-inflammatory *IL-1β*, *IL-8*, *TNF-α*, and *IL-12* [37].

In conclusion, LPS induced inflammatory response in the macrophages of Siberian sturgeon at 6 h, 12 h, and 24 h. Transcriptome sequence showed that DEGs significantly enriched in NOD-like receptor signaling pathway with pro-inflammatory cytokines up-regulation and multiple NLRC3-like down-regulation. Further study found that a total of 19 NLRs consisting of NLR-A, NLR-C, and other NLRs were mined from the transcriptome database of Siberian sturgeon, which lacked the PYD and B30.2. This study indicates that NLRC3-like is involved in the inflammatory response and provides the target to maintain host homeostasis when infected with bacteria.

## 4. Materials and Methods

### 4.1. Fish

The Siberian sturgeon (2.74 ± 0.53 kg) were purchased from Runzhao Fisheries Co., Ltd. (Chengdu, China). For a temporary period of two weeks, the fish were kept at 19.3 ± 0.2 °C and fed with a ratio of 2% of the total weight of commercial food at 9:00 and 16:00. The pH of aerated water was 7.3 ± 0.4, the concentrations of NH_3_ were less than 0.04 mg/L, and the dissolved oxygen was over 6.0 mg/L. All animal procedures were approved by the Animal Care and Use Committee of Sichuan Agricultural University.

### 4.2. Head Kidney Macrophages Culture and LPS Treatment

Head kidney macrophages culture was prepared as described by Zhu with modifications [38]. After the Siberian sturgeon were anesthetized with MS-222, the head kidney tissue was quickly dissected and placed into medium 1 on the ice. The 100-mesh cell sieve filtration was used to obtain the cell. Then, the cell suspension was added to 51% percoll, 400× *g*, at 4 °C, and centrifuged for 30 min. Moreover, the cells in the middle white layer were collected and counted. The cells were divided into the 24-well plate to incubate for 8 h, then added LPS solution to achieve 25 μg/mL and PBS as control. After 6 h, 12 h, and 24 h, the macrophages were collected.

### 4.3. Quantitative Real-Time PCR (qRT-PCR)

Total RNA was extracted from macrophages by RNAiso Plus (Takara, Dalian, China), and the cDNA was synthesized from 1 μg total RNA with the PrimeScriptTM RT Reagent Kit with gDNA Eraser (Takara, Dalian, China). *A. baerii*-specific *β-actin* and *GAPDH* primers served as the internal control to normalize the cDNA quantity for the sample. qRT-PCR of *IL-1β*, *TNF-α*, *IL-8*, and *TGF-β* was performed in a fluorescent quantitative instrument (Bio-Rad) by using the SYBR^®^ Premix Ex Taq^™^ II (Tli RNaseH Plus) (Takara, Dalian, China) in different times. Genes name, primers information, and product of qRT-PCR are listed in Table 3. The data were calculated using the comparative threshold cycle method (2^−ΔΔCT^).

### 4.4. RNA Isolation, mRNA Library Construction, and Sequencing

Total RNA was extracted from macrophages, and the concentration and purity of the RNA were detected by Nanodrop 2000 (NanoDrop Technologies, Wilmington, DE, USA). RNA integrity was detected by agar-gel electrophoresis and Agilent 2100 (Agilent, Beijing, China). Only high-quality RNA samples were used to construct the sequencing library.

The messenger RNA was isolated according to the polyA selection method by oligo (dT) beads and then fragmented by fragmentation buffer. Then, the double-stranded cDNA was synthesized using a SuperScript double-stranded cDNA synthesis kit with random hexamer primers. The synthesized cDNA was subjected to end-repair, phosphorylation, and ‘A’ base addition according to Illumina’s library construction protocol. Libraries were size selected for cDNA target fragments of 300 bp on 2% Low Range Ultra Agarose followed by PCR amplified for 15 PCR cycles. At last, the paired-end RNA-seq sequencing library was sequenced with the Illumina NovaSeq 6000 sequencer.

### 4.5. Transcriptome Processing and Assembly

The sequencing service was provided by Majorbio (Majorbio, Shanghai, China). The fastp (https://github.com/OpenGene/fastp) (accessed on 9 November 2018) was used to perform the quality control for the raw paired-end reads. Then, the clean reads were separately aligned to the sterlet reference genome (GCF_010645085.1) with the HISAT2 (http://ccb.jhu.edu/software/hisat2/index.shtml) (accessed on 6 August 2017). The StringTie (https://ccb.jhu.edu/software/stringtie/) (accessed on 21 April 2020) was used to map and assemble the reads of each sample.

### 4.6. Gene Annotation and Classification

The assembled transcriptome sequences were compared with six databases to obtain the annotation information: NCBI nonredundant (NR, https://ftp.ncbi.nlm.nih.gov/blast/db/) (accessed on 24 August 2020), Swiss-Prot (http://web.expasy.org/docs/swiss-prot_guideline.html) (accessed on 8 December 2019), Pfam (http://pfam.xfam.org/) (accessed on 27 September 2018), Clusters of Orthologous Groups of proteins (COG, http://www.ncbi.nlm.nih.gov/COG/) (accessed on 25 November 2020), Gene Ontology (GO, http://www.geneontology.org) (accessed on 28 June 2020) and Kyoto Encyclopedia of Genes and Genomes (KEGG, http://www.genome.jp/kegg/) (accessed on 1 July 2020).

### 4.7. Identification of Differentially Expressed Genes (DEGs) and Enrichment Analysis

RSEM (http://deweylab.biostat.wisc.edu/rsem/) (accessed on 14 February 2020) was used to quantify gene abundances, and the transcriptome and assembly results were compared. Differential expression analysis was performed using the DESeq2, and the unigenes with the |log2FC| ≥ 1 and *p* adjust <0.05 were defined as DEGs. GO functional enrichment and KEGG pathway analysis were performed by Goatools and KOBAS, respectively.

### 4.8. NLR Scan and Analysis

The local database of the genome of sterlet and paddlefish was constructed. The sequence containing the NACHT motif (PF05729.15) was obtained from Pfam, and all E-value < 10^−10^ genes were obtained by HMMER 3.0. After removing the variable clipping, the longest sequence was used for subsequent analysis. The local BLAST database of Siberian sturgeon transcriptome was constructed, and the NLRs of Siberian sturgeon were obtained by comparing the NLRs sequences of sterlet and paddlefish one by one. MEGA-11 was used for NLRs sequence alignment and phylogenetic tree construction. The Interactive Tree of Life (ITOL, https://itol.embl.de/) (accessed on 22 April 2021) was used for the visual process.

### 4.9. Statistical Analysis

All experimental data were expressed as mean ± standard error (mean ± SEM). The results were analyzed by SPSS 27.0 and graphed by GraphPad Prism 8.0. Statistical analysis was performed using a *t*-test. *p* values < 0.05 indicated significance.

## Figures and Tables

**Table 1:** Quality control of the sequence.

Sample	Raw Reads	Clean Reads	Clean Bases	Error Rate (%)	Q30 (%)	GC Content (%)
PBS_1	46,677,332	45,822,444	6.35 G	0.0255	93.80	46.64
PBS_2	44,472,856	43,771,700	6.06 G	0.0253	93.93	48.97
PBS_3	42,289,914	41,679,648	5.75 G	0.0253	93.97	47.31
PBS_4	43,448,798	42,727,782	6.32 G	0.0255	93.82	26.9
LPS_1	41,257,692	40,638,064	5.61 G	0.0251	94.22	47.36
LPS_2	43,363,986	42,547,796	6.30 G	0.0254	93.87	49.02
LPS_3	41,645,024	41,011,368	5.67 G	0.025	94.28	48.53
LPS_4	52,862,782	52,064,962	7.16 G	0.0251	94.20	47.94

**Table 2:** Summary of the annotations of unigenes.

Database	Unigenes	Percentage (%)
GO	28,060	78.67
KEGG	24,675	69.18
COG	32,495	91.10
NR	34,336	96.27
Swiss-Prot	32,148	90.13
Pfam	30,373	85.15
Total annotation	34,484	96.68
Total unigenes	35,668	100

**Table 3:** Genes name, primers information, and product of qRT-PCR.

Gene Name	Primer Sequence (5′-3′)	Actual Tm (°C)	Product (bp)
*IL-1β*-F	TGATGAACGAGCTGGATGGG	57.1	114
*IL-1β*-R	GCTGGGTCTGCGGTATGTAG		
*TNF-α*-F	TCGCCGGACTTCACAATAGG	58.9	114
*TNF-α*-R	GCTTGCTCGCCAGTTGTTTT		
*IL-8*-F	GGTGCAAATTCTCCCAGCAAA	61.4	107
*IL-8*-R	AACTCCACTCCCAAAGGAGC		
*TGF-β*-F	ATTCAGAACTATAAGACCCCCC	63.3	161
*TGF-β*-R	CGGAAGTCAATGTAAAGAGGC		
*β-actin*-F	GTTGGTATGGGACAGAAGGACA	61.4	105
*β-actin*-R	CCAGTTGGTAACAATGCCGT		
*GADPH*-F	TGTGGGCATCAATGGATTTGG	60.5	119
*GADPH*-R	ACACCATGTATTCCGGGTCAAT		

## Data Availability

The data presented in this study are available on request from the corresponding author.
